# Sustainable Materials for Sustainable Energy Storage: Organic Na Electrodes

**DOI:** 10.3390/ma9030142

**Published:** 2016-03-01

**Authors:** Viorica-Alina Oltean, Stéven Renault, Mario Valvo, Daniel Brandell

**Affiliations:** Department of Chemistry-Ångström Laboratory, Uppsala University, Box 538, 751 21 Uppsala, Sweden; Alina.Mihali@kemi.uu.se (V.-A.O.); Steven.Renault@kemi.uu.se (S.R.); Mario.Valvo@kemi.uu.se (M.V.)

**Keywords:** Na-ion battery, organic electrode material, disordered carbons, renewable materials

## Abstract

In this review, we summarize research efforts to realize Na-based organic materials for novel battery chemistries. Na is a more abundant element than Li, thereby contributing to less costly materials with limited to no geopolitical constraints while organic electrode materials harvested from biomass resources provide the possibility of achieving renewable battery components with low environmental impact during processing and recycling. Together, this can form the basis for truly sustainable electrochemical energy storage. We explore the efforts made on electrode materials of organic salts, primarily carbonyl compounds but also Schiff bases, unsaturated compounds, nitroxides and polymers. Moreover, sodiated carbonaceous materials derived from biomasses and waste products are surveyed. As a conclusion to the review, some shortcomings of the currently investigated materials are highlighted together with the major limitations for future development in this field. Finally, routes to move forward in this direction are suggested.

## 1. Introduction

The last decade has seen a dramatic increase in interest for energy storage systems, not least through the exponential growth of published research papers and patents on Li and Li-ion batteries. This interest has been driven by parallel needs for new storage solutions for intermittent renewable energy sources and for portable storage in the transport sector, which is currently dependent on fossil fuels to a very high degree. Novel sustainable storage solutions are necessary for future energy systems. However, lifecycle assessment studies have raised important issues regarding the overall environmental impact and resource depletion associated with Li-ion batteries (LIBs), where their competiveness can be challenged by other storage systems [1,2]. The manufacturing of conventional LIBs requires a large amount of non-renewable materials and high energy consumption during processing. Further, the rather limited existing recycling strategies are dependent on a large consumption of energy and toxic chemicals [3]. Lithium is not a very scarce element, but its resources are far from unlimited and the sensitivity in supply of the metal can certainly cause large price fluctuations, not least if demand will increase rapidly [4]. 

**Figure 1:**
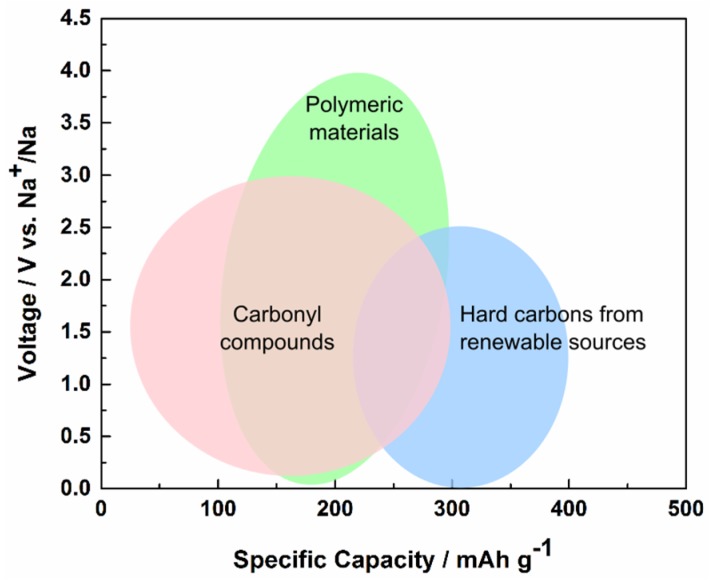
Average voltage *vs.* Na^+^/Na and capacity for some families of organic electrode materials.

For future battery chemistries, there are primarily two strategies suggested and employed to resolve this issue: either the Li-based systems are replaced by more abundant analogues such as Na, or the inorganic non-renewable materials are replaced by organic counterparts, which are—or can be—synthesized from biomass resources. From a perspective of materials resource sustainability, Na provides a better solution while the organic electrodes provide a better option regarding energy consumption and recycling. Both strategies have shown promising results and cell concepts, utilizing a broad range of active materials with a large variation in electrode potential and capacity (Figure 1), although significant developments are still required before widespread commercialization can occur. A few studies have also tried to combine these two approaches in organic Na-based chemistries, thereby creating a joint effort towards a truly sustainable battery. It is these contributions we set out to summarize in this review.

Although Li resources are extensive, the rapid demand from not only LIB applications but also future generation nuclear power plant materials, manufacturing goods such as glasses and ceramics, and pharmaceuticals are putting a strain on current reserves. For these particular reasons, it has been argued that Li might become the “new gold” [5]. In this perspective, Na is by comparison cheap, its common raw materials easily processed and the element is not affected by any significant limitation in geographical distribution. Na-based batteries attracted a lot of research and development interest during the 1970s and 1980s, but the applications were primarily limited to high-temperature devices for large-scale grid storage, such as the ZEBRA battery [6]. With the commercial success of the Li-ion battery from 1991, the Na-based counterparts were generally left aside. The last five years have, on the other hand, seen an increasing interest in low-temperature Na-ion batteries (SIBs), where a large number of intercalation materials analogous to those of LIBs have been explored. This has largely been a development driven by the abundance and cost of Na. 

The Na^+^/Na electrochemical potential is slightly higher than Li^+^/Li, thereby generating a theoretically lower voltage in the resulting cells. Since Na is a heavier element than Li, its compounds display a lower specific gravimetric capacity if compared on the basis of simple ion substitution. This is, however, of less relevance for true battery systems, where alkaline metal electrodes are less likely to be implemented using today’s electrolyte systems, and where more severe restrictions apply to both voltage and capacity compared to what is typically caused by the alkaline metal ions. In fact, the transition metal ions used in cathodes, the chemical instability of the electrolyte, the rapid ageing experienced by the electrodes, and the limited temperature interval of operation all pose perhaps larger challenges to the battery systems. Nevertheless, SIBs have so far displayed lower capacities and voltages compared to LIBs, at least for half-cell formats. The larger radius of the Na ion also generates a different and possibly more limited selection of intercalation hosts and larger volume expansions during cycling, which can in turn contribute to more pronounced ageing. On the other hand, significant improvements can be expected in the near future, in addition to the other advantages associated with SIBs. Among these is the possibility of employing the low-cost and lightweight metal Al as a current collector on the anode side instead of Cu which is currently used for LIBs. As a matter of fact, copper contributes significantly to the price of the ultimate devices and also causes a remarkable emission of greenhouse gases due to its expensive manufacturing process [7]. Thus, while Li alloys easily with Al at low voltages *vs.* Li^+^/Li, Na does not form any alloy or intermetallic compound with Al, thereby yielding a mechanically and chemically stable current collector for anodic materials which also lowers the cost and environmental impact of the final device.

The vast majority of compounds for LIB and SIB electrodes are typically inorganic, *i.e.*, extracted from ores and therefore non-renewable. The standard LIB electrode materials are lithium transition metal oxides or phosphates (e.g., LiFePO_4_, LiMn_2_O_4_, LiCoO_2_, LiNi_0.5_Mn_1.5_O_4_, Li_4_Ti_5_O_12_, *etc.*), or lithiated graphite (LiC_6_). These materials generally demand toxic chemicals and strong acids during extraction, while recycling requires high temperature combustion, strong acid treatment and highly energy-demanding electrolysis—a process where all the Li is lost. On the other hand, the last decade has seen significant advancement regarding organic materials as potential electrodes for LIBs. Thus far, due to inherent problems with low volumetric energy density, poor conductivity and solubility in most common electrolytes, these efforts have mainly been limited to academic research, but the field is nevertheless expected to approach a commercial breakthrough in terms of lifespan, cost, and energy density for these materials [8]. For example, organic radical batteries are anticipated to reach the market very soon [9]. 

Although the use of organic electrode materials for LIB applications is not new, this domain was, until a few years ago, only sporadically studied due to the superiority in performance of inorganic compounds (e.g., higher volumetric energy density). The electroactivity of certain organic compounds has been recognized for a long time, in particular redox-active polymers like polyaniline, polyacetylene, polypyrrole, *etc.* [10]. After pioneering work on LIBs, such polymers were also investigated for SIBs by Shacklette *et al.* (polyacetylene, poly(*p*-phenylene), and polypyrrole [11,12,13]). More recently, the development of organic radical batteries has demonstrated design possibilities for flexible devices [14]. Limitations are usually associated with poor electrical conductivity of the radical polymers and with the fact that such conjugated polymers involve mainly the ingress of the electrolyte anion (and not Li^+^). Organodisulfides [15] have also been recognized as promising organic cathode materials for LIBs, however, despite various efforts to overcome slow kinetics and dissolution phenomena, these drawbacks have prevented further development in this direction. Furthermore, conjugated carbonylated molecules, which are abundant in nature, have been extensively explored as potential anode materials since Poizot *et al.* proposed the use of dilithium rhodizonate, Li_2_C_6_O_6_, a lithiated version of a material that can be obtained from corn [8,16]. These classes of organic LIB electrode materials can also be well explored for Na counterparts.

**Figure 2:**
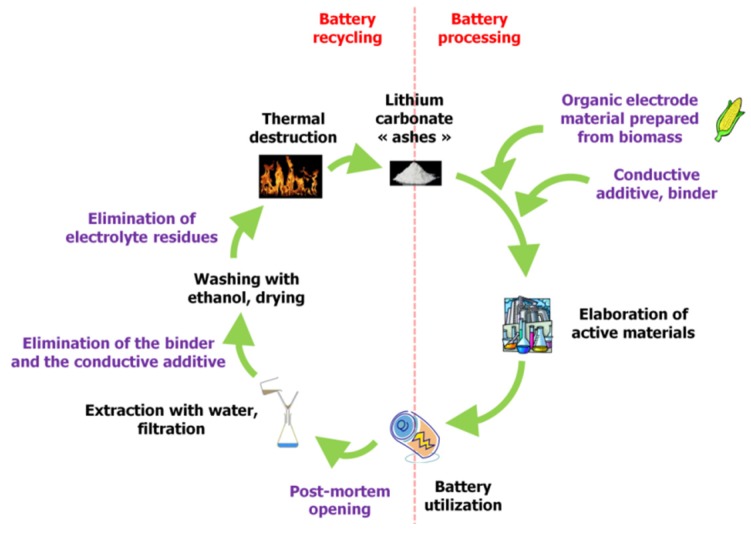
Idealized lithium recycling process of spent batteries made of water-soluble organic electrode materials issued from biomass.

In this context, it should also be recognized that utilization of organic electrode materials significantly contributes to more viable recycling possibilities. Battery electrodes can be thermally destroyed using low-temperature processes, and Li_2_CO_3_ can been recovered from the resulting ash using extraction in water and ethanol [17]. If using organic materials from biomass, this closes the entire loop for the battery lifecycle (Figure 2).

Another category of materials that can store large quantities of Li or Na (or other cation type) salts is different porous carbonaceous materials. This is a rapidly growing field for electrochemical storage materials, where high-temperature carbonization is generally utilized for the synthesis of the host compounds [18]. This is especially true for SIBs, where so-called “hard” carbons have proven significantly more useful than the ordered LIB standard graphite anode. Accordingly, disordered carbons represent a promising class of materials for use in these batteries, especially at the anode side. A wide range of different starting materials have been investigated, of which a significant degree originates from biomass resources in one way or another. As such, these have a clear potential to contribute to sustainable storage materials, not least if the carbonization temperature is kept reasonably low. 

If the research areas of SIBs and organic electrodes can be considered young, the field of organic Na-ion batteries (OSIBs) is clearly still in its infancy. However, this field is rapidly emerging, and the many achievements reported by different research groups around the world have now reached a critical level where some general conclusions can possibly be drawn. Indeed, a multitude of recent reviews exist on sodium-ion batteries [7,19,20,21], in addition to a large number of contributions summarizing the field of organic electrodes [10,15,22,23,24]. However, to the best of our knowledge, no review focusing on OSIBs has been published so far. Moreover, it can also be argued that Na is considered to be especially relevant for organic electrodes. The larger ionic radius of Na^+^ as compared to Li^+^ constitutes a limitation for the number of intercalation materials available, but the rather large voids present in most of the organic electrode materials can well provide solid-state transport paths for Na. Therefore, this area can offer fruitful synergistic effects on the atomic level. 

## 2. Carbonyl Insertion Compounds

**Figure 3:**
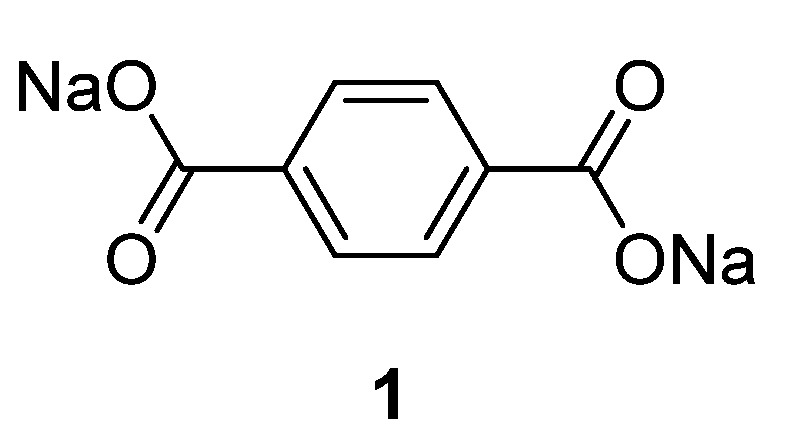
Molecular structure of disodium terephthalate, Na_2_C_8_H_4_O_4_ **1**.

Table 1 summarizes the key properties of the OSIB insertion compounds explored so far in the literature. Perhaps the most popular compound investigated as an organic material for the anode part in SIBs is disodium terephthalate **1** (Figure 3). Several variations of this compound have been investigated: from the pure compound to electrodes employing metal-oxide (Al_2_O_3_) coatings generated by atomic layer deposition (ALD) for 20 and 50 cycles, to the exploration of different substituents such as –NH_2_, –Br, and –NO_2_ attached to the backbone [25,26,27]. Disodium terephthalate **1** presents a low sodium insertion voltage of 0.29 V *vs.* Na^+^/Na and a high reversible capacity of 250 mAh·g^−1^ with good cycleability. When the performance of this compound was tested between 0.1 and 2 V, a pattern emerged with voltage drops to 0.7 V during the initial discharge process followed by a plateau at 0.29 V, after which a second drop at 0.23 V could be observed. On the charge side, the plateau is situated at 0.56 V and the coulombic efficiency is 50.3%. This behavior changed during subsequent cycles and the low coulombic efficiency was explained by the large irreversible capacity caused by Solid Electrolyte Interphase (SEI) layer formation, seen as a sloping region around 0.7–0.29 V in the cycling data. It has also been demonstrated that if this compound is coated with a protective metal-oxide layer, the performance will improve due to the properties of the surface coating, which allows easier formation of an SEI layer from the reductive decomposition of the electrolyte during the first discharge.

When disodium terephthalate derivatives were investigated, it was noticed that different cycling performances could be obtained depending on the type of substituent on the benzene ring. For example, for the cases of Br and NH_2_ (**2** and **3**), different capacity retentions, 300 mAh·g^−1^ and 200 mAh·g^−1^, respectively, were observed due to the electron withdrawing effect of one group (Br), while the other (NH_2_) exhibits electron donating properties. However, despite the different types of substituents, both substituted species display one-phase reactions while the non-functionalized compound exhibits a two-phase reaction [27]. The nitro group was also examined (**4**), and this presents yet another type of behavior, due to the group storing two additional Na^+^ ions. This compound displays a good capacity during the first cycle, 302 mAh·g^−1^, but the capacity fades with each cycle and a somewhat unstable cycling behavior was also observed. Similar to how the performance is influenced by the addition of substituents, region-isomerism of this compound also contributes to a variation in properties. For example, disodium isophthalate displays a lower capacity value, 220 mAh·g^−1^, and a sloping voltage profile which indicates a one-phase reaction, similar to the compounds with Br and NH_2_ substituents [27].

Disodium terephthalate **1** in the form of nano-sheets was also investigated [26]. The nanosheets could be obtained by a straightforward liquid-phase strategy. From the electrochemical performance, it is visible that the nanosheets present an enhanced capacity in comparison to the bulk material. They exhibit not only a one-step desodiation mechanism in comparison to the two-step mechanism of the bulk material, but also enhanced electronic conductivity and cation migration. According to the results obtained by the authors, the nanosheet morphology exhibits a larger reversible capacity of 248 mAh·g^−1^ in comparison to the bulk material (only 199 mAh·g^−1^) and better cycling performance. After 100 cycles, the nanosheets display a capacity of 105 mAh·g^−1^ *vs.* 60 mAh·g^−1^ for the bulk material.

Magnesium terephthalate **5** has also been investigated as an anode material for sodium-ion batteries [28]. This represents an innovative approach, where a different cationic type than the intercalating ion is introduced to stabilize the salt structure. Although the anhydrous material results in rather poor performance, the analogous compound containing two molecules of water generated a capacity of 114 mAh·g^−1^ in the first cycle, decreasing to 95 mAh·g^−1^ for the next 50 cycles.

**Figure 4:**
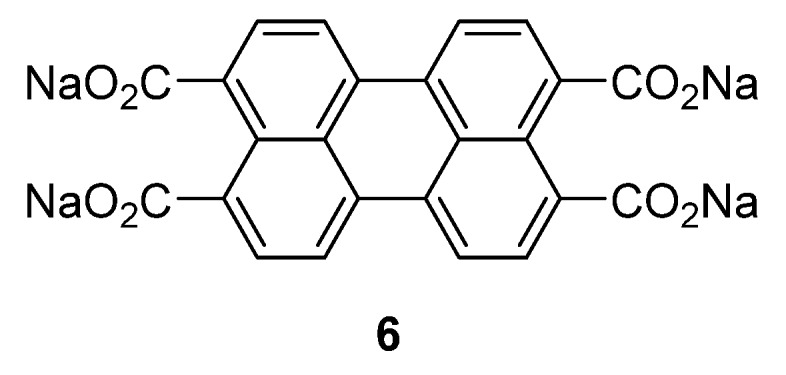
Molecular structure of sodium perylene-3,4,9,10-tetracarboxylate **6**.

If the aromatic core is increased with larger conjugated perylene rings, the chemical stability can be improved through decreased solubility in the (organic) electrolyte [29]. An example of this type of compound was investigated by Zhao *et al.* [25]: sodium salt of perylene-3,4,9,10-tetracarboxylic acid **6** (Figure 4). This compound was tested in the potential range of 0.3–2.8 V *vs.* Na^+^/Na at a constant current of 25 mA·g^−1^, producing charge-discharge plateaus at voltages of 0.6/0.4 V and a reversible capacity of 100 mAh·g^−1^ with good cycling stability.

**Figure 5:**
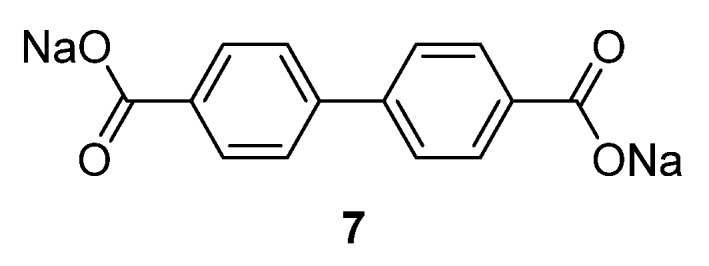
Molecular structure of 4,4’-biphenyldicarboxylate sodium salt **7**.

4,4′-biphenyldicarboxylate disodium salt **7** (Figure 5) and the monosodium salt were also investigated as an anode material for SIBs [30]. These two salts show a good electrochemical performance with a reversible capacity of 200 mAh·g^−1^ at approximately 0.5 V *vs.* Na/Na^+^ with very little capacity fading over 150 cycles. Even at a high rate of 3.74 A·g^−1^ (20 C; *i.e.*, 10 cycles/h), the compounds still possess decent capacity retention, with a capacity of approximately 100 mAh·g^−1^. 

**Figure 6:**
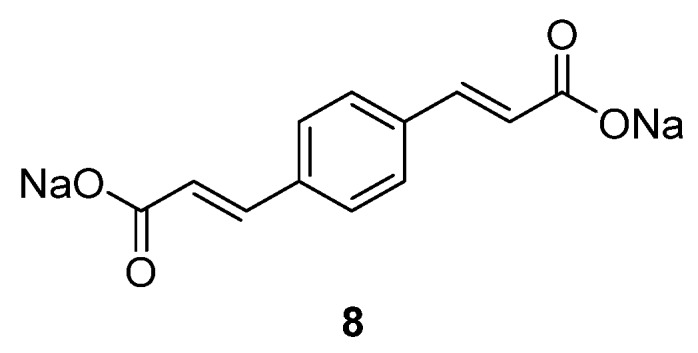
Molecular structure of disodium benzenediacrylate **8**.

Sodium organic materials investigated within our group include disodium benzenediacrylate **8** (Figure 6) [31]. This compound presents a good initial capacity of 177.7 mAh·g^−1^, but decreases to ~50 mAh·g^−1^ after 20 cycles. If the C-rate increases, the capacity increases relative to its Li homologue, which loses capacity once the cycling rate is increased. Moreover, the Na compound displays a higher initial coulombic efficiency of 91% in the initial cycle, which increases to ~95% after 40 cycles. Even though the compound requires improvement in terms of capacity retention, it represents one option for OSIBs and highlights the challenges associated with the transition from lithium-based to sodium-based chemistries [47,48].

**Figure 7:**
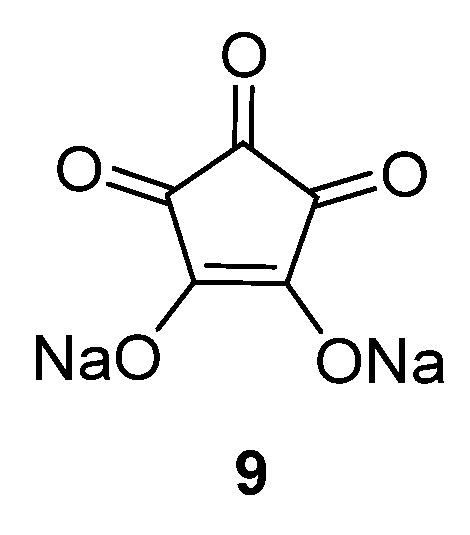
Molecular structure of disodium salt of croconic acid **9**.

Croconic acid disodium salt **9** (Figure 7) was also studied as an anode material for SIBs [32]. This compound is based on Na insertion on carbonyl groups that are connected by a conjugated carbon matrix. The carbonyl groups are redox centers, which enable electrochemical reactions to occur. The pristine material delivers a high capacity of 246.7 mAh·g^−1^ in the first cycle; however, the capacity decreases significantly during subsequent cycles. If the particle size of this compound is reduced to submicrometer dimensions, or if a graphene oxide wrapping of the particles is applied, the capacity can temporarily be increased. However, after 100 cycles the capacity reduces to ~100 mAh·g^−1^.

**Figure 8:**
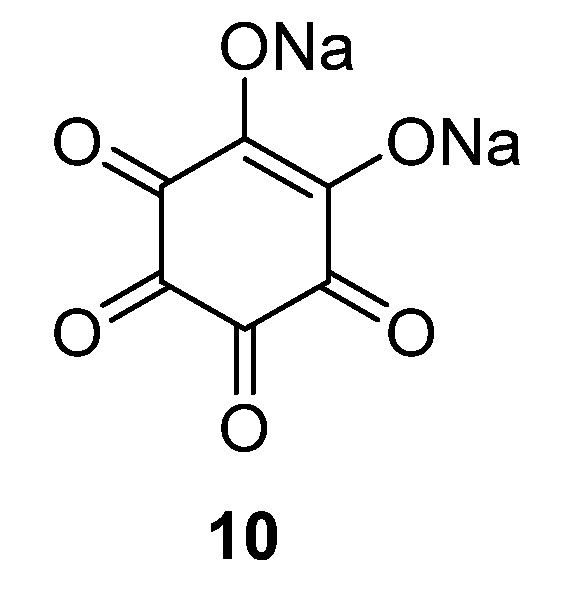
Molecular structure of disodium rhodizonate **10**.

Although focus of OSIB research has clearly been on anode materials, the cathode side has not been completely abandoned. In fact, research in this area has been fruitful during the past years. However, it is debatable whether several of these reduction-functional compounds are more suitable as high-potential anodes. One example of an OSIB cathode compound is disodium rhodizonate **10** (Figure 8). This displayed good results even without conductive additive, which is rare for an organic electrode material [33]. At a potential of 2.18 V, the compound shows a good performance with a capacity of 170 mAh·g^−1^. Different potential regions and binders (such as PTFE binder instead of the conventional PVdF) have also been employed for this material in order to improve its performance.

Humic acid **11**, considered a representative of natural polymers, has also been proposed as an anode material for SIBs [34]. This compound can be extracted from woods, soils and coals. When cycled against sodium, the compound displays a reasonably high capacity of 244 mAh·g^−1^ during the first cycle and a reversible capacity of 208.3 mAh·g^−1^. The promising behavior of this compound can be ascribed to the fact that the polymeric structure will inhibit the dissolution process of the active material into the electrolyte. Moreover, the compound provides reasonable capacity values at high cycling currents, for example 60 mAh·g^−1^ at 400 mA·g^−1^. However, due to the presence of several functional groups (*ortho-* and *para*-quinones, carboxylic acid, Schiff bases, *etc.*) and its polymeric nature, its actual redox mechanism is difficult to establish.

**Figure 9:**
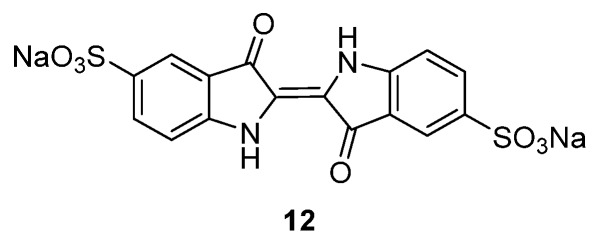
Molecular structure of indigo carmine **12**.

Indigo carmine **12** (Figure 9) is another compound that has been considered for both Li and Na systems [35]. In the case of SIBs, the compound is able to insert two Na^+^ ions on the carboxylate groups. The performance of this compound in sodium-ion cells is promising, with a capacity of 105 mAh·g^−1^ in the first cycle and a constant value of 95 mAh·g^−1^ for 40 cycles, in the voltage range 1.5–3.0 V *vs.* Na^+^/Na. Moreover, the electrodes present a coulombic efficiency of almost 100% in the subsequent cycle, suggesting a high reversibility of this material to store both Na and Li. In comparison to the low-molecular-weight organic materials which suffer from poor cycle performance due to the dissolution of the active material in the electrolyte, the indigo carmine molecule has a very low solubility in ordinary organic solvents due to the peripheral polar sulfonate groups. 

**Figure 10:**
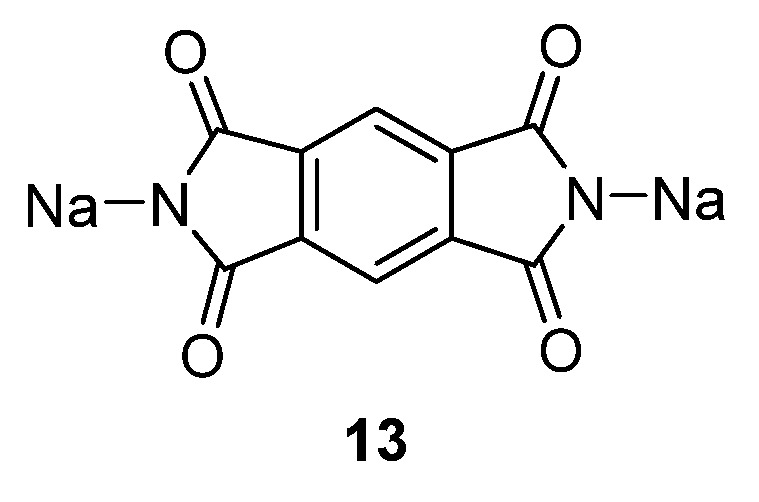
Molecular structure of disodium pyromellitic diimidate **13**.

Another compound investigated by us is disodium pyromellitic diimidate **13** (Figure 10) [36]. A similar problem of decreasing capacity could also be seen in this case. The compound exhibits excellent capacity in the first cycle which reduces in subsequent cycles to ~90 mAh·g^−1^. This capacity can, however, still be considered acceptable for a non-optimized OSIB material.

**Scheme 1:**
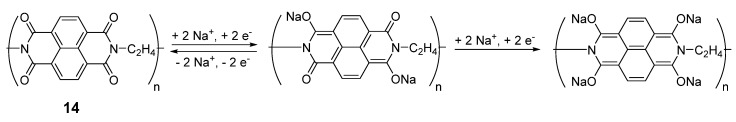
Molecular structure of 1,4,5,8-naphthalenetetracarboxylic dianhydride derived polyimide **14** and the redox mechanism of this compound.

Similar types of structures can be found in polymers such as polyimides. One such example can be synthesized from 1,4,5,8-naphthalenetetracarboxylic dianhydride and ethylene diamine [37]. Each monomer of this compound **14** can de/insert two Na^+^ ions (Scheme 1), delivering a capacity of 140 mAh·g^−1^ at an average potential of 2 V *vs.* Na^+^/Na with high coulombic efficiency and excellent stability over 500 cycles with a capacity retention of 90%. The macromolecular structure of the compound inhibits the problems associated with solubility in the electrolyte, enhancing performance and stability. The compound was also cycled against Na_3_V_2_(PO_4_)_3_/C and Na_4_Fe(CN)_6_/C cathodes to form 1.2 V sodium full-cells. Considering the sum of anode and cathode materials, these cells gave capacities of 75 mAh·g^−1^ and 70 mAh·g^−1^, respectively. Despite possessing four carbonyls, the reduction on both **13** and **14** is reversible on only two of them, as a complete reduction of the diimide system leads to irreversible degradation in both lithium- and sodium-ion battery systems.

Perylene based (dianhydride-based) polyimides (PIs) were also studied as cathode materials for SIBs [38]. The PTCDA (perylene 3,4,9,10-tetracaboxylic dianhydride)–based PI **15** delivers a specific power of 20–99 kW·kg^−1^ and specific energy of 285 Wh·kg^−1^ by retaining 87.5% of the initial capacity over 5000 cycles. The use of the perylene-based conjugated structure is an advantage in comparison with 1,4,5,8,-naphthalenetetracarboxylic **14** or pyromellitic **16** compounds due to higher working potential and better stability during cycling.

Some of the materials investigated for SIBs were also included in full cells, either against an inorganic material or in an all-organic sodium-ion cell. One of the examples found is *N,N′*-diamino-3,4,9,10-perylenetetracarboxylic polyimide (PI-IMI) **17 [39]**. First, the PI-IMI was investigated in a half-cell configuration between 1.5 and 3.5 V. The polymer generated a capacity of 126 mAh·g^−1^ and capacity retention of ~90% after 50 cycles. When this material was cycled *vs.* the previously discussed disodium terephthalate **1**, the full-cell provided an average potential of 1.35 V and a capacity of 73 mAh·g^−1^; calculated based on the weight of both anode and cathode active materials.

Disodium terephthalate **1** was also cycled in a full cell using Na_0.75_Mn_0.70_Ni_0.23_O_2_ as the cathode [49]. Once the cell was assembled, a 3.6 V battery could be obtained with an initial capacity of 257 mAh·g^−1^ based on the anode weight, and displayed only slight capacity decay to 238 mAh·g^−1^ during the first 50 cycles. The cell was cycled at a current rate of 20 mA·g^−1^ (~C/13; *i.e.*, 1 cycle per 26 h) and, even though the coulombic efficiency was 43% for the first cycle, a value of 99% could be achieved during subsequent cycles.

Quinones have also been examined as cathode materials [40]. The substitution with halogens on benzoquinone increases the redox potential without increasing the molecular weight. The high electronegativity of the halogen groups draws electrons from the molecule, thereby increasing the redox potential. Benzoquinones substituted with chlorine and fluorine atoms have also been investigated as organic electrode materials for SIBs. While the fluorine derivative **18** forms NaF by irreversibly detaching the F from the C_6_F_4_O_2_ structure, the chlorine compound **19** can be considered a promising battery material displaying de/insertion of Na^+^ ions, if the problem associated with its high solubility in common organic electrolytes is solved. Investigations of Na insertion into C_6_F_4_O_2_ electrodes by XPS analysis has demonstrated that no noticeable shift in the F_1s_ peak is observed upon discharge. The peak simply loses intensity, and a new peak at ~684 eV corresponding to NaF appears, indicating that the F irreversibly detaches from the structure and forms NaF. The formation of this compound is not clearly understood, and the authors speculate that it is related to the higher polarizability of the Cl as compared to F. In the charge process there are no further changes to the F peaks, indicating that the formation of NaF is irreversible. The Cl compound renders a capacity of 160 mAh·g^−1^ with fast capacity decay in the following cycles, reaching a value of ~30 mAh·g^−1^ after 20 cycles. By combining this compound into a composite with ordered mesoporous carbon, the capacity could be improved to ~60 mAh·g^−1^ after 20 cycles. 

**Figure 11:**
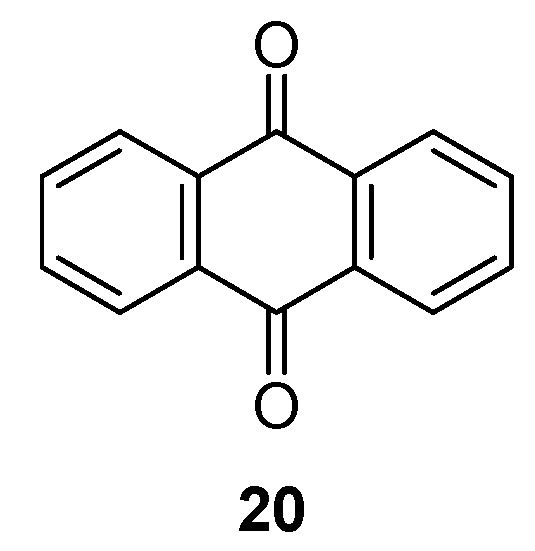
Molecular structure of 9,10-anthraquinone **20**.

A strategy designed to tackle the dissolution phenomenon of quinones was given by Chen *et al.* [41]. The highly soluble 9,10-anthraquinone **20** (Figure 11) was encapsulated in a nanostructured porous carbon, CMK-3, in a 1:1 mass ratio. The composite electrode material cycled in a high viscosity ether-based electrolyte was less likely to dissolve **20** than common carbonate-based electrolytes. Using a high concentration of 4 M sodium triflate (NaTFS) in tetraethylene glycoldimethyl ether (TEGDME) electrolyte, **20** displayed an initial discharge capacity of 214 mAh·g^−1^ (average redox potential 2.8 V *vs.* Na^+^/Na) which dropped to 200 mAh·g^−1^ after 10 cycles and then stabilized for moderate capacity losses (190 mAh·g^−1^ and 88% capacity retention after 50 cycles).

**Figure 12:**
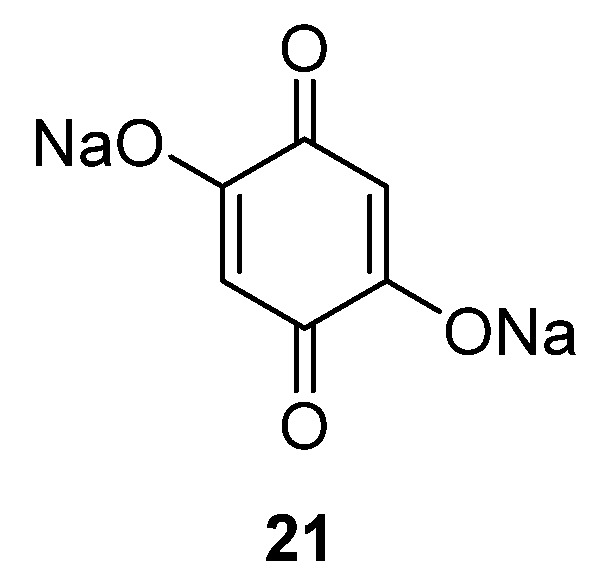
Molecular structure of 2,5-dihydroxy-1,4-benzoquinone **21**.

Alternatively, quinone materials could be designed to possess at least two permanent negative charges in order to avoid dissolution, such the case for 2,5-dihydroxy-1,4-benzoquinone **21** (Figure 12) [42]. After a capacity loss from 398 to 265 mAh·g^−1^ in the first two cycles due to irreversible capacity, this compound still provides a capacity of 231 mAh·g^−1^ after 50 cycles (87% capacity retention from the second cycle) even when cycled in a carbonate-based electrolyte (1 M NaClO_4_ in EC/DMC). However, the negative charges present in the vicinity of the carbonyls increase the electronic density of the redox functions, causing the average potential to drop to 1.3 V *vs.* Na^+^/Na compared to other quinones. For this material, its crystalline structure is a framework consisting of Na–O polyhedral layers (which both improves the Na^+^ conduction pathway and stores additional sodium ions) and organic π-stacked benzene layers for electron diffusion and as a redox center [43]. 

**Scheme 2:**
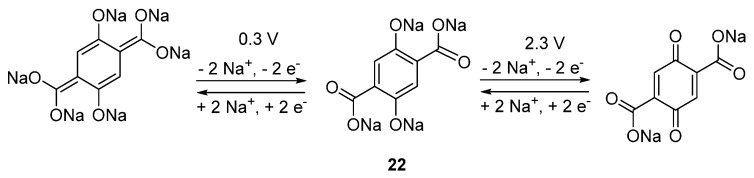
Molecular structure for tetrasodium salt of 2,5-dihydroxyterephthalic acid **22** and the redox mechanism for sodium insertion in an all-organic rocking-chair cell.

The investigation of the tetrasodium salt of 2,5-dihydroxyterephthalic acid **22** (Scheme 2) at both high potentials, such as 1.6–2.8 V, and in parallel at low potentials, such as 0.1–1.8 V *vs.* Na^+^/Na, generates an all-organic ”rocking-chair” OSIB [44]. The material contains two different groups, each with a different Na de/insertion mechanism. On one side, the enolate groups will receive or expel Na^+^ at 1.6–2.8 V, whereas on the other side the carboxylate groups will perform this reaction at 0.1–1.8 V. This material can deliver capacities of 180 mAh·g^−1^ if cycled in half-cells against Na metal, and when in a full cell deliver an average working potential of 1.8 V and a capacity of ~150 mAh·g^−1^ after 100 cycles. Here the capacity is calculated based on the mass of the negative electrode, Na_4_C_8_H_2_O_6_, with capacity contributions from the Super P. Once again, this demonstrates the diversity and tunable properties of the organic molecules.

Finally, carbonyl materials have generated a lot of interest as OEMs for SIBs during the past four years. In particular, their low volumetric density as compared to inorganic materials could be seen as an advantage in terms of providing sufficient sodium diffusion pathways in the crystalline framework. Many inorganic materials intended as negative electrodes suffer from limited sodium diffusion due to the fact that the diffusion pathways are adapted for smaller lithium ions. As a consequence, OEMs can be seen as a valid alternative for applications where the volumetric energy is not essential. Most notably, the performance of sodium terephthalate as an anode is superior to most investigated inorganic materials.

## 3. Schiff Bases

**Figure 13:**
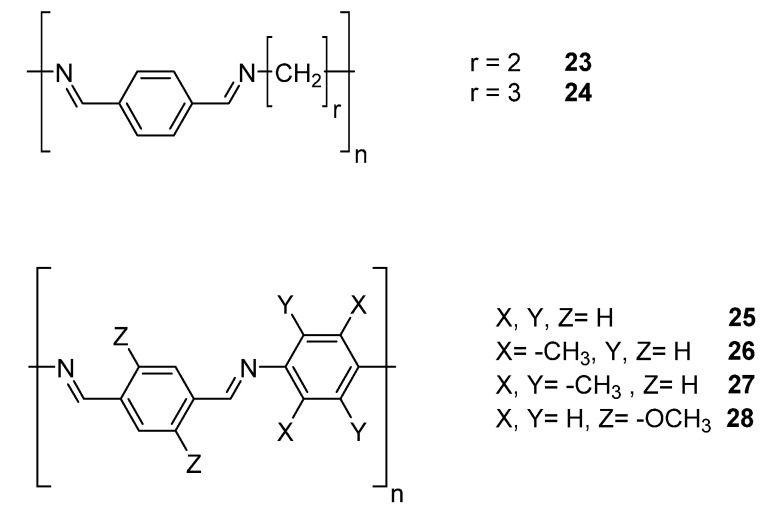
Molecular structures of polymeric Schiff bases **23–28**.

Another variety of organic materials that have been explored for SIBs are based on the Schiff base functionality R^1^HC=NR^2^. For these compounds, the electrochemical activity can be expected from the existence of the C=N double bond, which has been proven in polarographic studies at low potentials in organic electrolytes. Polymeric Schiff bases (Figure 13) have also been investigated as negative electrode materials for SIBs, all of which have the repeating unit –N=CH–Ar–HC=N– in common. At least six different polymers based on Schiff bases **23**–**28** have been synthesized and studied in sodium (half) cells, demonstrating that they can deliver capacities from around 150 up to 180 mAh·g^−1^ [45].

**Figure 14:**
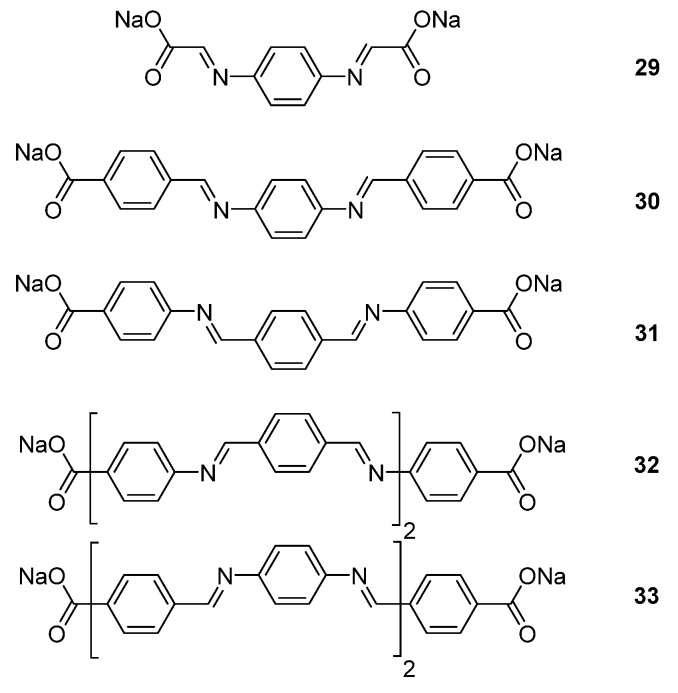
Molecular structure of oligomeric Schiff bases **29–33**.

Oligomeric Schiff bases (Figure 14) can be synthesized using a condensation reaction of di/aldehydes and aromatic amines. Five types of bases **29**–**33** have been synthesized this way, all from the same precursor but with variation in oligomer length or orientation of the –C=N– bond [46]. They display relatively stable cycling with capacities from 50 to 300 mAh·g^−1^ and an operating voltage of <1.2 V *vs.* Na^+^/Na. Their performances have been optimized by increasing the number of active units along the total length of the chain.

## 4. Sodium Insertion on Carbon–Carbon Double Bonds

**Scheme 3:**
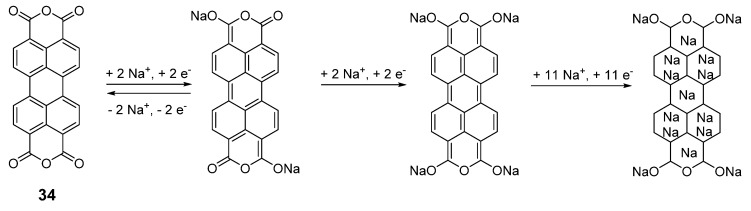
Proposed reduction mechanism in PTCDA **34** according to reference [51].

Similar to what has been published for the LIB system [50], sodium insertion on carbon–carbon double bonds has occasionally been reported. For instance, for perylene 3,4,9,10-tetracarboxylic dianhydride (PTCDA) **34**, after the expected reduction of the carbonyls, a reduction at very low potential was observed that could not be attributed to the conductive additive or SEI layer formation [51]. Overall, a total of 15 sodium ions were found to be inserted in the structure after the initial discharge (corresponding to 1017 mAh·g^−1^) (Scheme 3). However, such a mechanism is also associated with very large hysteresis, poor energy efficiency and severe capacity fading for reasons that are still not well understood (decomposition of the material, electrolyte decomposition, contact loss with current collectors due to severe volume expansion, *etc.*, has been speculated).

Similar performances for PTCDA were later observed by Wang *et al.* [52]. They also investigated the behavior of the sodium salt of perylene-3,4,9,10-tetracarboxylic acid **6** at more reducing potentials than in previous work (down to 0 V as compared to 0.3 V *vs.* Na^+^/Na for reference [29]). However, unlike the former compound, **6** displays a specific capacity that can be explained by the redox mechanism of the carbonyls solely. 

The combination of large hysteresis and unusually high specific capacity has also been observed cases where the redox mechanism is not explained, such as the polybithiophene (which is structurally equivalent to polythiophene) prepared by Zhu *et al.* [53]. When tested in a sodium half-cell as an n-type material, the initial reduction delivered a 1187 mAh·g^−1^ discharge capacity that dropped to ~400 mAh·g^−1^ after 40 cycles, which is likely due to significant sodiation of the unsaturated bonds.

It is clear that this mechanism is interesting from a fundamental perspective due to the unusually high specific capacity theoretically obtainable which compensates for the low energy density of OEMs. However, practical applications might be limited by sluggish kinetics, energy efficiency and limited cyclability and will depend on further development on this topic which is still in its infancy.

## 5. Anion Insertion Compounds

**Scheme 4:**
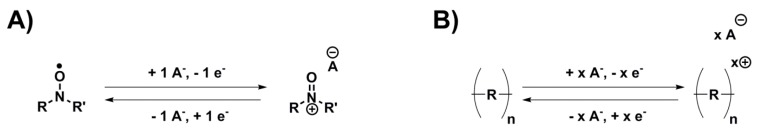
Reversible redox organic functional groups working as anion insertion for sodium-ion batteries: (**A**) nitroxide and (**B**) conducting polymer (if R is a conjugated or aromatic system).

Anion insertion, often called *p*-doping when referring to a polymeric material, is a mechanism where the electron loss during oxidation is counterbalanced with an uptake of one anion from the electrolyte, and the reverse during reduction, making it independent of sodium ions and their associated slow kinetics. Although few investigations have been published on anion insertion for SIBs (Table 2), two different sub-classes reacting according to this mechanism have still been investigated (Scheme 4):

- Nitroxide/nitroammonium chemistries, which are usually associated with the fastest kinetics obtained for organic electrode materials despite limited conductivity and capacity.
- Conducting polymers, which combine good conductivity and kinetics but have limited energy density.

The only example to date using the nitroxide/nitroammonium redox couple for a SiB application is the work from Dai *et al.* who implemented a material already known for its performance in organic radical batteries (ORBs): poly[norbornene-2,3-*endo,exo*-(COO-4-TEMPO)_2_] **35** [54]. Due to its poor conductivity, **35** was mixed with a large amount of the high surface area additive vapor growth carbon fiber (VGCF) in a 1:2 w:w (weight:weight) ratio of **35** and VGCF. The polymer was shown to uniformly cover the surface of the fibers in the composite electrode, and a high average potential of 3.4 V *vs.* Na^+^/Na was obtained. However, both cyclic voltammetry (CV) and galvanostatic cycling measurements display significant irreversible capacity during the first cycle and a mere 25% of coulombic efficiency was obtained after an initial 296 mAh·g^−1^ charge. As a consequence of limited coulombic efficiency (63% on the second cycle), the reversible capacity drops from 75 to 48 mAh·g^−1^ after 50 cycles (64% initial capacity retention). The authors explained this limitation with the formation of a thick passive layer on the anode and with sodium dendrite formation.

Redox conducting polymers for SIBs have mostly been investigated by the group of Yang *et al.* [53,55,56,57,59]. They reported a study on an aniline/*ortho*-nitroaniline copolymer **36** [55]. Although the authors claimed that the polymer obtained from their synthesis has a leucoemeraldine oxidation state, they also mentioned the presence of quinone diimine units along with spectroscopic evidence, which therefore corresponds to an emeraldine oxidation state for their material. This copolymer displays overall good performance with a sloping electrochemical profile centered at 3.2 V *vs.* Na^+^/Na when cycled galvanostatically. The charge capacity increases from an initial 181 mAh·g^−1^ to 195 mAh·g^−1^ at the tenth cycle and remains rather stable over 50 cycles.

**Scheme 5:**
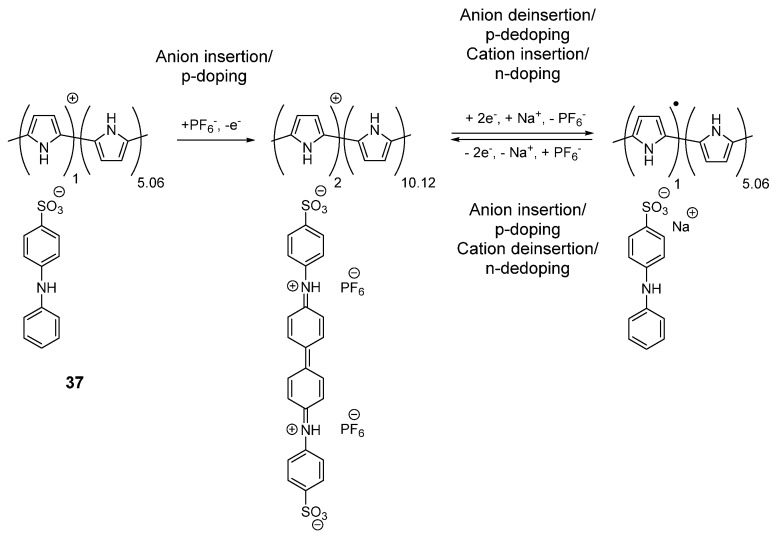
Redox mechanism of diphenylamine-4-sulfonate (DS)-doped polypyrrole **37**.

The same research group has also compared polypyrrole and diphenylamine-4-sulfonate (DS)-doped polypyrrole **37**; the latter showing clearly enhanced performances [56]. It displays a sloping electrochemical profile between 3.7 and 2.4 V *vs.* Na^+^/Na with low polarization and an initial capacity of 115 mAh·g^−1^, which is three times higher than for pristine polypyrrole. Moderate capacity loss is observed after 50 cycles. Curiously, the redox mechanism is explained by the authors as joint anion and cation insertion due to the presence of DS anions. For this compound, the first charge is an anion insertion (in this case PF_6_^−^) where the electron is lost on the DS anion (Scheme 5). Although not clearly explained by the authors, diphenylamine-4-sulfonate usually undergoes dimerization under such conditions [60]. After this activation step, the reduction takes place through the reverse process and parallel sodium insertion and electron uptake on the polypyrrole chain. This mechanism was justified with Fourier-Transform Infrared (FTIR) spectroscopy and Inductive Coupled Plasma (ICP) analysis where PF_6_^−^ was detected only in the charged material and Na^+^ in the discharged polymer. A similar work with parallel anion insertion on a conducting polymer and sodium insertion was also published using Fe(CN)_6_^4−^-doped polypyrrole [59].

**Scheme 6:**
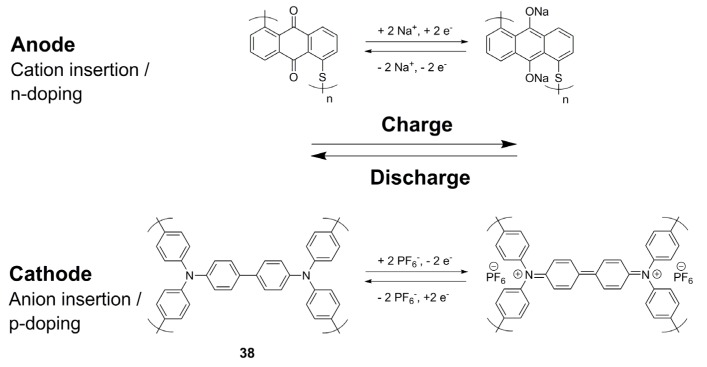
Electrochemical reactions in a full cell using poly(anthraquinonyl sulfide) as negative and polytriphenylamine **38** as positive electrode material.

Another example is polytriphenylamine **38** [57]. This material has a high average potential (3.6 V *vs.* Na^+^/Na) and displays stable capacity even at high C-rate, that is, 88 mAh·g^−1^ at 2000 mA·g^−1^ (20 C). It was used as a cathode in an all-organic full cell where the poly(anthraquinonyl sulfide anode material was reacting according to sodium insertion (average potential: 1.6 V *vs.* Na^+^/Na). Such a cell, employing an appropriate cathode/anode balance, displays a practical capacity above 150 mAh·g^−1^ for 500 cycles at a 1600 mA·g^−1^ rate (8 C) (Scheme 6). 

The polymeric framework consisting of triazine and benzene rings **39** is another case of a material able to insert both anion (between 2.8 and 4.1 V *vs.* Na^+^/Na) and cation (between 2.8 and 1.3 V *vs.* Na^+^/Na) during cycling [58]. It displays a specific energy of 500 Wh·kg^−1^ and retains 80% of its initial capacity at a current density of 0.1 A·g^−1^.

## 6. Sodiated Hard and Soft Carbons from Renewable Resources

Significant attention has been paid to the use of disordered carbons as possible negative electrodes for SIBs, and it is quite attractive to obtain these materials directly from renewable sources. The key properties of these materials are summarized in Table 3. The use of disordered carbons as anodic materials in SIBs is dictated by the fact that graphite, differing from Li^+^ in LIBs, cannot insert Na^+^ reversibly [61,62] in combination with standard electrolytes. However, Na^+^ insertion can take place in the presence of solvent co-intercalation phenomena in diglyme-based electrolytes [63]. Two types of disordered carbons have been applied for this purpose: soft and hard carbons. The distinction between these forms is subtle and is still under debate. Typically, soft carbons are characterized by a disordered structure and have the property of being graphitizable at higher temperatures, e.g., >2800 °C. In contrast, while they still possess a disordered structure similar to that of soft carbons, hard carbons cannot be transformed into graphite even at very high temperatures. Hard carbons are also particularly sensitive to their synthesis conditions and typically consist of two domains, carbon layers (graphene-like) and micropores, which are generated between disordered carbon layers (*i.e.*, not properly stacked as in graphite). 

The first disordered carbon material for SIBs produced by means of a renewable source was a type of hard carbon (HC) prepared via glucose carbonization at 1000 °C [64]. This material could deliver a reversible capacity of *ca.* 300 mAh·g^−1^ at a ~35 μA·cm^−2^ (C/80). After preliminary studies discussing the similarities of the insertion mechanisms for lithium and sodium in such carbonaceous materials [64,65,66], several other investigations were pursued in this direction. For example, a template-based approach relying on hydrothermal carbonization employed an aqueous dispersion of poly(styrene) latexes and d-glucose to form hollow carbon nanospheres (HCNS) via a two-step synthesis process [67]. A thin layer of hydrothermal carbon was first obtained around the latex during the hydrothermal decomposition of glucose at 180 °C and the material subsequently underwent non-oxidative pyrolysis at 1000 °C to remove the template and generate the hollow spheres. The specific surface area of the HCNS was 410 m^2^·g^−1^, which likely caused a noticeable irreversible capacity loss (*i.e.*, ~59%) during the first cycle performed at a current density of 50 mA·g^−1^ with a 1 M NaClO_4_ electrolyte in PC. The same test performed with the current density was increased to 100 mA·g^−1^ after the first ten cycles yielded a reversible capacity of about 160 mAh·g^−1^ after 100 cumulative cycles. 

Sucrose has also become another popular renewable source for preparing hard carbons. For example, sucrose carbonized at 1300 °C displayed reversible capacities approaching 300 mAh·g^−1^ at a current density of 25 mA·g^−1^ using 1 M NaClO_4_ electrolyte in PC with a slight addition of FEC [68]. Electrolyte optimization was also carried out on hard carbons derived from sugar pyrolysis at 1100 °C according to a synthesis method similar to that of Stevens *et al.* [64]. The latter was employed as a benchmark material for negative electrodes in SIBs and its performance was optimized via an electrolyte formulation based on 1 M NaClO_4_ (or NaPF_6_) in EC_0.45_PC_0.45_DMC_0.1_ [69]. 

Since the early studies in this field, it has been clear that the electrochemical performance of the resulting Na-ion cells are highly dependent on the specific area of these carbons and on possible impurities present on their surfaces. These properties have an influence on the formation mechanisms and subsequent growth of the SEI layer. In fact, one of the main problems for these materials is the large irreversible capacity loss observed during the first cycle due to SEI generation. This issue is still under intense study, where a low specific surface area is an important target for these carbonaceous materials and where additives are certainly required to stabilize their electrochemical performance during prolonged cycling. In a recent study, sucrose was used in combination with graphene oxide (GO) to obtain a very low surface area hard carbon [70] showing an initial coulombic efficiency of 83% and a stable capacity retention of about 280 mAh·g^−1^ for 300 cycles at 20 mA·g^−1^. Sucrose has been adopted also for the synthesis of hard carbon spherules (HCS), which were further coated by a layer of soft carbon via toluene pyrolysis [71]. Such treatment also resulted in an improved initial coulombic efficiency of 83%. The HCS exhibited interesting cycling performances with capacities up to 290 mAh·g^−1^ after 100 cycles at 30 mA·g^−1^ (C/10), with an overall capacity retention of 93% for the materials obtained at the highest temperature of 1600 °C.

Nonetheless, glucose and sucrose also represent important sources of energy, and, consequently, are fundamental from a nutritional point of view for human kind. From this perspective, it can be argued that many other alternative syntheses should be made available to prepare disordered carbons in a more sustainable way than by burning carbohydrates at very high temperatures under inert gas atmospheres. The carbonization process can for example be performed on waste materials and/or alternative biomasses, which typically have little use and run the risk of ending up in landfills, thereby creating other issues for their recycling and/or incineration processes. In this context, three main approaches have been pursued for the preparation of disordered carbons to be utilized in rechargeable Na-ion (and Li-ion) batteries:

- Carbonization of fresh biomass.
- Pyrolysis of spent biomass.
- Controlled carbonization of industrial organic waste materials.

Carbonization of fresh biomass can be considered attractive when the selected type of biomass is highly abundant, easily renewable within a short time-frame and, more importantly, when it does not interfere with food provision resources. The amount of CO_2_ and other greenhouse gases directly (and indirectly) liberated during their carbonization processes, as well as during possible further treatments, should be assessed carefully. Since the resulting carbonaceous materials should eventually be of ”battery-grade” quality, subsequent chemical treatments will most likely be employed thereby causing additional emissions. 

Some disordered carbons, which have been obtained by pyrolysis of fresh biomasses at different temperatures, are, for example, those derived from peat moss [72]. Peat moss is an earth-abundant wild plant that possesses a particular cellular structure. This property, together with the high degree of cross-linking found for the polymers in its cell walls, contribute to the formation of ordered pseudo-graphitic structures (PGS) with large interspacing between the graphene layers, thus facilitating sodium insertion and de-insertion during the electrochemical processes. A post-pyrolysis activation treatment in air at 300 °C further promotes the generation of a hierarchical porosity that can boost the overall electrochemical performance. Although the initial coulombic efficiencies for the first cycle have remained limited for all carbonized specimens (*i.e.*, below 65%), a capacity of about 255 mAh·g^−1^ could be achieved after 210 cycles in a sodium half-cell with an applied current density of 0.1 A·g^−1^ for 200 cycles (during the first ten cycles, a capacity of 298 mAh·g^−1^ was obtained at 0.05 A·g^−1^).

A similar route employed recently used chemically crushed wood cellulose fibers [73] to produce porous hard carbons. The overall surface area of the carbonized products was significantly reduced by a pre-treatment with 2,2,6,6-tetramethylpiperidine-1-oxyl (TEMPO), which for a sodium half-cell, led to a coulombic efficiency of 72% in the first cycle and a stable capacity of 196 mAh·g^−1^ for 200 cycles at 0.1 A·g^−1^. The way in which such oxidative pre-treatment can unzip hollow wood fibers, producing densely packed ribbon-like structures with a moderate surface area, is clearly advantageous to improve the reversibility of the corresponding anodic material. 

Another recent approach applied for the fabrication of porous, free-standing and binder-free carbon electrodes [74] relied on the pyrolysis of the cap skin tissue from ripe Portobello mushrooms. For the pyrolysis products obtained above 900 °C, an attractive morphology consisting of an interconnected network of carbon nanoribbons with hierarchical porosity was found and enabled extensive cycling of such as-synthesized electrodes in Li-based half cells. The carbonized materials displayed a high degree of self-activation upon cycling and practical capacities almost comparable to those obtained from conventional laminated graphite electrodes, reaching 260 mAh·g^−1^ after 700 cycles at a 17 mA·g^−1^ (C/5) rate. Their initial coulombic efficiency, however, remained limited below 50%, even after additional treatment. This route clearly demonstrates the benefits of exploiting the natural microstructures and fibers that exist in this edible and highly popular precursor. 

From a sustainability perspective, utilization of spent biomass is more attractive than that of fresh counterparts, since the biomass has first been employed for some main purpose and can then be re-utilized in a spent state, which normally is of little or no use. Indeed, even though pyrolysis is not an environmentally friendly process by default, a carbonization step can transform such waste biomasses into a valuable product, which in turn, can be utilized in negative electrodes for both Li- and Na-ion batteries. However, careful evaluation of the combustion gases and other possible sources of pollution must be taken into account and analyzed in terms of the efficiency for the ultimate carbon content that can be obtained. Nevertheless, if the pyrolysis were carried out properly, it would be possible to develop, in principle, convenient incineration protocols to save part of the thermal energy released in the process.

A variety of examples can be found on disordered carbons for negative electrodes in SIBs via pyrolysis of spent biomass. For example, shaddock (*i.e.*, pomelo) peels were utilized as precursors for carbonization processes in a few studies [75,76]. In an early investigation [76], the shaddock peels were also treated with H_3_PO_4_ and subsequently pyrolized at 700 °C in N_2_. The activation step contributed to create a larger surface area for the ultimate product, which displayed an extensive three-dimensional (3D) connected porous structure. However, this also caused a very low initial coulombic efficiency of 27%. Despite this limiting factor, such activated porous carbons yielded a decent capacity of 181 mAh·g^−1^ after 220 cycles at a current density of 0.2 A·g^−1^ in Na-half cells. 

Subsequent work [75] performed on pomelo peels relied merely on a one-step pyrolysis in N_2_ within the temperature range from 800 to 1400 °C. The absence of any additional treatment had a positive impact on the initial coulombic efficiency for the pyrolysis products, which exhibited values approximately between ~63% and ~70%. Capacities as high as 430 mAh·g^−1^ were achieved at an applied current of 30 mA·g^−1^. Further, significant capacity retention of 352 mAh·g^−1^ was attained after 200 cycles at 50 mA·g^−1^ for the hard carbons produced at 1200 °C. Such a value corresponded to a reversibility of 97.5% compared to the first reversible capacity (~361 mAh·g^−1^). Nonetheless, good cycling performance was maintained over 300 cycles at 0.2 A·g^−1^, with typical capacities approaching 160 mAh·g^−1^ for the carbons synthesized at 1000 °C. These attractive features were attributed to the particular morphology of the pyrolytic products and the large interlayer distance for their graphene-like sheets, which facilitated Na^+^ insertion and extraction. 

**Scheme 7:**
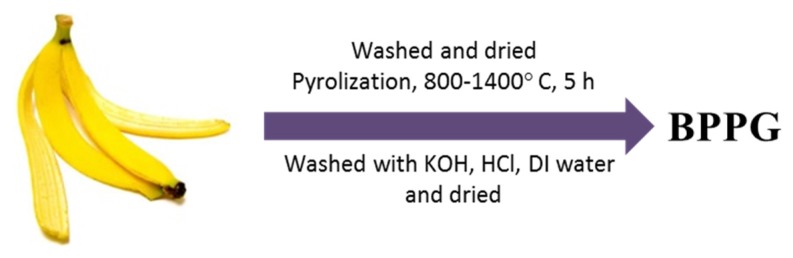
Synthesis procedure of BPPG (banana peel pseudo-graphite).

Carbonized banana peels have also been proposed as high-density anodes for both Li- and Na-ion batteries (Scheme 7) [77]. This type of spent biomass is appealing as a carbon precursor due to its large worldwide consumption and its scarce economical value as organic waste. Pyrolysis of banana peels’ was conducted at temperatures between 800 and 1400 °C in an argon atmosphere, and some of the resulting pseudo-graphitic samples were further activated in air at 300 °C. The overall electrochemical performance of these electrodes was remarkable upon testing in both Li- and Na-half cells. The latter was ascribed mainly to an expanded intergraphene spacing for Na^+^ insertion/extraction, a generation of easily accessible nanopores for metal filling at low voltages and a significant amount of defects in the graphene planes contributing to cation adsorption at higher potentials. Capacities as high as 355 mAh·g^−1^ were obtained after ten cycles in Na-half cells at a current density of 50 mA·g^−1^. Good capacity retention of nearly 300 mAh·g^−1^ was displayed after 300 cycles at 0.1 A·g^−1^. Even longer cycling, with capacities around 205 mAh·g^−1^, was reported after 600 cycles at 0.5 A·g^−1^. The irreversible capacity loss during the first cycle was reduced with increasing carbonization temperature (from 63% to 73% retention for 800 °C and 1400 °C, respectively), and the resulting specific surface areas of the products were low to moderate. Interestingly, the same materials outperformed graphite in terms of both gravimetric and volumetric capacities upon cycling in Li-half cells, with pronounced Li storage at low voltage, but with lithium metal plating in the pores of the structures. Stable capacity retention was obtained for all cases, though the initial coulombic efficiency was limited to values around or below 60%.

**Scheme 8:**
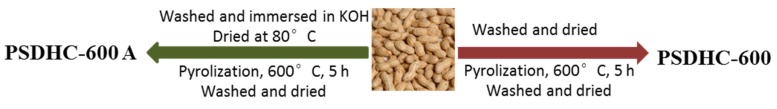
Synthesis procedure of porous PSDHC-600 (peanut shell derived hard carbon obtained by pyrolysis at 600 °C) and porous PSDHC-600 A (peanut shell derived hard carbon obtained by pyrolysis at 600 °C, previously treated with KOH solution).

A similar approach for both SIBs and LIBs was reported using peanut shells as a precursor for the synthesis of hard carbons with enhanced cycle lives (Scheme 8) [78]. Interestingly, the carbonization temperature, which gave the best results in terms of electrochemical performances, was 600 °C, thus consuming less energy than in the previous examples. In this case, an activation stage for the initial biomass was also utilized, where the peanut shells were immersed in an aqueous solution of 7 wt.% KOH before pyrolysis. This treatment greatly enhanced the electrochemical performance for the resulting material with respect to Li^+^ and Na^+^ storage. The coulombic efficiency for the first cycle was limited for both activated and untreated samples (*i.e.*, ~30%) upon cycling in Na-half cells at a current density of 0.25 A·g^−1^. Nonetheless, stable capacities around 190 mAh·g^−1^ and 130 mAh·g^−1^ could be achieved at this current density for the activated and untreated specimens, respectively, after 400 cycles. Furthermore, the activated sample displayed good capacity retention upon cycling at 1 A·g^−1^, with a final value of ~130 mAh·g^−1^ after 3000 cumulative cycles. In comparison, the electrochemical behavior of the activated sample was better than that of the untreated one upon testing in Li-half cells. A capacity close to 475 mAh·g^−1^ was reported for the activated carbon after 400 cycles at 1 A·g^−1^, whereas the untreated sample approached 315 mAh·g^−1^ under the same conditions. A test conducted at 5 A·g^−1^ on the activated carbon yielded a final capacity of about 310 mAh·g^−1^ after 10,000 cycles, although the initial coulombic efficiency was below 40%.

Lignin has also been utilized as a feedstock material for the preparation of cheap carbon fibers with adequate properties for anodic materials in both Li- and Na-ion batteries [79]. Lignin is attractive since it is a byproduct of the pulp and paper industry. It thus represents an appealing renewable biopolymer for these purposes. Recent advances in the synthesis of lignin precursors and their conversion into carbon fibers have led to good electrochemical performances for these carbonaceous materials in both Li- and Na-ion cells.

Finally, carbonization of industrial organic waste materials is also attractive and adds value to the end products, since the hard carbons are prepared from harmful organic waste that cannot immediately be recycled in economical ways. Materials like non-degradable plastic waste severely pollute soil and waterways and accumulate in landfills. Their incineration is even more hazardous, since noxious polluting gases are released during combustion. In this sense, achieving convenient and non-harmful carbonization is challenging. Unlike spent biomasses, which can still find more or less sustainable ways of being re-incorporated into the carbon cycle with limited environmental impact, industrial organic wastes pose a severe threat to the environment from the early stages of synthetic preparation of their original materials. 

The fact that artificial polymers such as polyvinyl chloride (PVC) or polystyrene (PS) have been employed as potential precursors for the preparation of hard carbons for SIBs is of importance in this context. For example, hard carbons produced via direct pyrolysis of PVC in the form of both particles and nanofibers was reported recently for such purposes [80]. The PVC nanofibers were fabricated via an electrospinning technique and were subsequently subjected to the same pyrolytic conditions (*i.e.*, 600–800 °C in Ar) as for the PVC particles. The results for hard carbons derived from nanofibers are quite promising. An initial coulombic efficiency close to 70% and a stable capacity of 211 mAh·g^−1^ were obtained in Na-half cells after 150 cumulative cycles mostly performed at 12 mA·g^−1^. The electrospinning step appeared to make the difference in the performance of the two forms of pyrolized precursors. Although this methodology represents an elegant approach, e.g., by providing perspectives on potential applications for PVC waste functionalization, the process relies only on the direct pyrolysis of a commercial PVC precursor. No assessments regarding the gases released upon carbonization and/or the vapors of the solvents used in the nanofiber fabrication were made in this study, and the environmental footprint of the entire process can therefore not be quantified. 

**Scheme 9:**
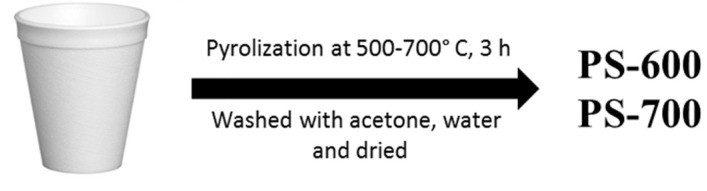
Synthesis procedure of PS-600 and PS-700 (disordered carbons obtained by carbonization of polystyrene at 600 °C and 700 °C, respectively).

In contrast, a recent study on the synthesis of disordered carbons via plastic waste recovery has focused on polystyrene (PS) directly obtained from waste cups (Scheme 9) [81]. This approach is certainly appealing for achieving better sustainability for the carbonization process, and several relevant properties for efficient recycling are also clearly addressed in this study. The final performance of the resulting materials proved to be not as good as those for the alternative materials described above. However, the possibility of obtaining disordered carbons (DCs) via the carbonization of PS cups under high pressure was evaluated accurately. A temperature of 700 °C was reported to offer the best electrochemical performances in Na-half cells with a capacity of 116 mAh·g^−1^ after 80 cycles at a current density of 20 mA·g^−1^. The initial coulombic efficiency remained rather low (53%), likely due to the high specific surface area found for the carbon products (88 m^2^·g^−1^). Nonetheless, such an attractive route to transform waste PS plastic into functional materials for more sustainable SIBs deserves proper attention.

## 7. Conclusions

The development of potentially sustainable organic materials as active electrodes for SIBs has seen remarkable growth of interest in the last five years. The accumulated knowledge on ambient-temperature batteries over the last few decades and the chemical analogies between lithium and sodium has instigated tremendous progress of the field in a rather short time. While still in an infant stage, the large variety of organic structures allows an ease in tuning the targeted properties (average potential, theoretical specific capacity, and kinetics) depending on the redox function and molecular structure. However, the examples summarized above also show that it is still challenging for such materials to achieve parallel energy/power density and cyclability. There is still plenty of room for improved OSIBs and to couple these devices with low CO_2_ footprint, low cost production via biomass, as well as through material recycling. OSIBs could well serve as a potentially cheap and disposable energy storage system in our daily lives for several applications, complementarily to high-energy LIBs.

A few general conclusions can be made from the above analysis. First, successful strategies need to be employed for capacity retention in the resulting OSIB. The previous examples show that inorganic coatings or polymerization approaches can provide solutions to these kinds of problems. Second, the capacity is inherently linked to the number of functional groups present in the samples. Unsaturated carbons can in this context provide such functionality by introducing large possibilities for sodiation without adding mass to the active materials. If the reversible sodiation processes can be stabilized without material degradation, this could certainly be most promising in terms of energy density of the devices. Third, it is clear that most material development has been for anode compounds, and so efforts on developing cathode OSIB materials are sorely needed. Here, organic LIB materials can certainly continue to serve as an inspiration. Interestingly, the development of inorganic SIB materials has displayed a reverse trend, *i.e.*, better choices for cathodes than for anodes are available, which suggests that hybrid organic/inorganic SIB cells might be more realistic chemistries in the near future. Fourth, biomass-derived carbonaceous materials have displayed promising properties in terms of coulombic efficiency, capacity, and cycle stability. Proper lifecycle assessment and emission analyses are necessary to clarify the possible environmental advantages of using this approach for SIB electrode materials fabrication, as well as possible recycling strategies (e.g., also when utilized with organic counter-electrodes). Nevertheless, the combination of renewable organic materials together with the high abundance of sodium raw materials provides an appealing concept. The latter is enough for promoting a continuous advancement, and research in this field should certainly be appreciated.

## Figures and Tables

**Table 1:** Structures and electrochemical performances of carbonyl insertion compounds and Schiff bases for OSIBs.

Structure	First Cycle Capacity (mAh·g^−1^)	Practical Capacity (mAh·g^−1^)	Average Potential (V *vs.* Na^+^/Na°)	Electrolyte	Reference Number
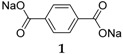	375	250	0.395	1 M NaFSI in EC:DEC	[25]
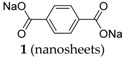	250	105	0.5	1 M NaClO_4_ in EC:DEC	[26]
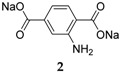	300	200	0.4	0.8 M NaClO_4_ in EC:DEC	[27]
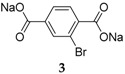	450	300	0.5	0.8 M NaClO_4_ in EC:DEC	[27]
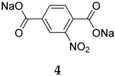	300–500	150–300	1.1	0.8 M NaClO_4_ in EC:DEC	[27]
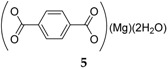	120	90	0.7	1M NaClO_4_ in PC:DMC	[28]
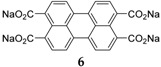	110	~100	0.5	NaPF_6_-saturated in EC:DEC	[29]
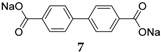	220	~200	0.5	0.8 M NaClO_4_ in EC:DEC	[30]
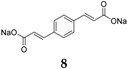	177	50	0.65	1 M NaFSI in EC:DEC	[31]
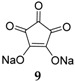	~280	~100	1.3	1 M NaClO_4_ in EC:DMC	[32]
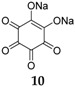	270	90	2.18	1 M NaClO_4_ in PC	[33]
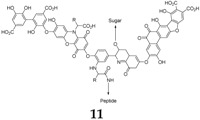	240	133	0.9	1 M NaPF_6_ in PC:DMC	[34]
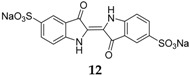	110	90	1.8	1 M NaTFSI in butylene carbonate	[35]
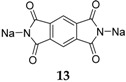	200	90	1.2	0.8 M NaPF_6_ in PC	[36]
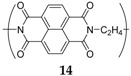	150	~140	2	1 M NaClO_4_ in EC:DEC	[37]
	120	110	1.8	1 M NaPF_6_ in EC:DMC	[38]
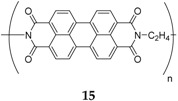	110	100	1.9	1 M NaPF_6_ in EC:DMC	[38]
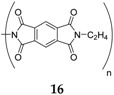	148	137	2	1 M NaPF_6_ in EC:DMC	[38]
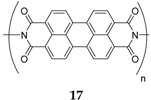	126	~105	2.45	1 M NaPF_6_ in PC	[39]
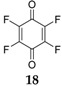	~140	No charge capacity	2.9	1 M NaClO_4_ in EC:PC:DMC	[40]
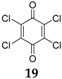	150	~30	2.72	1 M NaClO_4_ in EC:PC:DMC	[40]
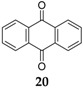	214	190	1.8	4 M NaTFS in TEGDME	[41]
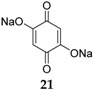	398	240	1.3	1 M NaClO_4_ in EC:DMC	[42]
	288		1.3	0.8 M NaPF_6_ in PC	[43]
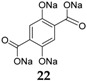	207	184	0.3	1 M NaClO_4_ in EC:DMC	[44]
	200	183	2.3		
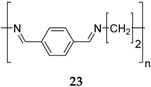	200	150	0.7	1 M NaFSI in Me-THF	[45]
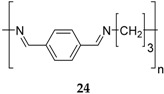	100	~50	~0.5	1 M NaFSI in Me-THF	[45]
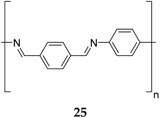	250	180	0.8	1 M NaFSI in Me-THF	[45]
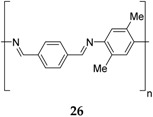	–	–	0.6	1 M NaFSI in Me-THF	[45]
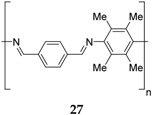	–	–	–	1 M NaFSI in Me-THF	[45]
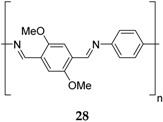	–	–	1.0	1 M NaFSI in Me-THF	[45]
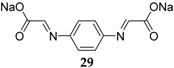	~600	50	1.0	1 M NaFSI in Me-THF	[46]
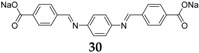	~500	220	0.9	1 M NaFSI in Me-THF	[46]
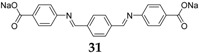	~400	100	0.8	1 M NaFSI in Me-THF	[46]
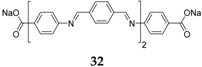	~500	150	0.9	1 M NaFSI in Me-THF	[46]
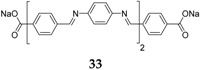	~500	215	0.9	1 M NaFSI in Me-THF	[46]

**Table 2:** Anion insertion compounds for OSIBs.

Structure	First Charge Capacity (mAh·g^−1^)	Practical Capacity (mAh·g^−1^)	Average Potential (V *vs.* Na^+^/Na°)	Electrolyte	Reference Number
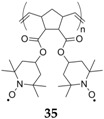	296	75 to 48 over 50 cycles	3.4	1M NaClO_4_ in EC/DMC 1/1	[54]
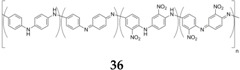	181	195 to 173 over 50 cycles	3.2	1M NaPF_6_ in EC/DMC/DEC 1/1/1	[55]
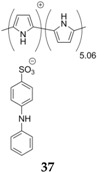	105	115 to 95 over 50 cycles	2.9	1M NaPF_6_ in EC/DEC 1/1	[56]
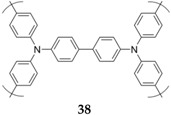	105	96 to 88 over 200 cycles	3.6	Saturated NaPF_6_ in DME/DOL 1/1	[57]
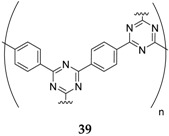	235	235 to 200 over 40 cycles	2.8	1M NaClO_4_ in PC	[58]

**Table 3:** Comparison of some relevant features for various types of amorphous-like carbon cycled in Na-half cells under different galvanostatic conditions.

Carbon Structure	Precursor for Carbonization	Carbonization Temperature (°C)	Specific Area (m^2^·g^−1^)	Electrolyte Components	Q_charge_/Q_discharge_ 1^st^ Cycle (%)	Capacity at 100^th^ Cycle (mAh·g^−1^)
HCNS	Glucose	1000	410	NaClO_4_, PC	≈41	≈160 @ 100 mA·g^−1^
HC	Sucrose	1300	n.r.	NaClO_4_, PC/2% FEC	≈85	≈180 @ 25 mA·g^−1^
HC	Sucrose/GO	1100	5	NaPF_6_, EC/DEC	≈83	≈230 @ 20 mA·g^−1^
HCS	Sucrose/Toluene	1600	n.r.	NaClO_4_, EC/DEC	≈84	≈290 @ 30 mA·g^−1^
PGS *	Peat moss	1400	92	NaClO_4_, EC/DEC	≈60	≈210 @ 100 mA·g^−1^
HC *	Pomelo peels	700	1272	NaClO_4_, EC/PC	≈27	≈180 @ 200 mA·g^−1^
HC	Pomelo peels	1200	83	NaClO_4_, EC/DEC	≈68	≈190 @ 200 mA·g^−1^
PGS *	Banana peels	1400	62	NaClO_4_, EC/DEC	≈73	≈290 @ 100 mA·g^−1^
OCP *	Cellulose fibers	1000	126	NaClO_4_, EC/DEC	≈72	≈200 @ 100 mA·g^−1^
HC *	Peanut shells	600	706	NaClO_4_, EC/DEC	≈30	≈210 @ 250 mA·g^−1^
CNF	PVC fibers	700	n.r.	NaPF_6_, EC/DEC	≈70	≈220 @ 12 mA·g^−1^
DC	PS cups	700	88	NaClO_4_, EC/DEC	≈53	≈115 @ 20 mA·g^−1^^§^

**Legend:** n.r. means not reported; * activation of the raw (or formed) material; @ precedes the current value used in the respective studies; ^§^ at cycle no. 80 (only 80 cycles reported).

## Abbreviations

The following abbreviations are used in this manuscript:

ALD: atomic layer deposition
BPPG: banana peel pseudo-graphite
CNF: carbon nanofibers
CV: cyclic voltammetry
DEC: diethyl carbonate
DMC: dimethyl carbonate
DC: disordered carbon
DS: diphenylamine-4-sulfonate
EC: ethylene carbonate
FEC: fluoroethylene carbonate
FT-IR: Fourier-transform infrared
GO: graphene oxide
HC: hard carbon
HCNS: hollow carbon nanospheres
HCS: hard carbon spherules
ICP: inductive coupled plasma
LIB: Li-ion batteries
OCP: oxidized carbon paper
OSIB: organic Na-ion batteries
PC: propylene carbonate
PGS: pseudo-graphitic structure
PI: Polyimide
PI-IMI: *N,N′*-diamino-3,4,9,10-perylenetetracarboxylic polyimide
PS: Polystyrene
PTCDA: perylene-3,4,9,10-tetracaboxylic dianhydride
PVC: polyvinyl chloride
SEI: Solid Electrolyte Interphase
SIB: Na-ion batteries
TEGDME: tetraethylene glycoldimethyl ether
TEMPO: 2,2,6,6-tetramethylpiperidine-1-oxyl
TFS: Triflate
VGCF: vapor growth carbon fiber
